# Harmonizing color measurements in dentistry using translucent tooth-colored materials

**DOI:** 10.1186/s12903-024-03935-1

**Published:** 2024-02-02

**Authors:** Rubens Nisie Tango, Claudia Ângela Maziero Volpato, Karina Félix Santos, Paulo Francisco Cesar, Rade Dušan Paravina

**Affiliations:** 1 School of Dentistry, Institute of Science and Technology, Department of Dental Materials and Prosthodontics, Sao Paulo State University, Avenida Eng. Francisco José Longo, 777, São José dos Campos, Sao Paulo 12245320 Brazil; 2 Department of Dentistry, The Federal University of Santa Catarina School of Dentistry, R. Delfino Conti, Florianópolis - Santa Catarina, 1240, 88040-535 Brazil; 3 Faculty of Dentistry, Department Biomaterials and Oral Biology, University of Sao Paulo, Av. Professor Lineu Prestes, Sao Paulo - Sao Paulo, 2227, 05508000 Brazil; 4 Department of Restorative Dentistry and Prosthodontics, University of Texas School of Dentistry at Houston, 7500 Cambridge St, Houston, TX 77054 USA

**Keywords:** Color measurement, Spectrophotometry, Calibration, Psychophysics

## Abstract

**Background:**

Since color measurements are relative, the discrepancy among different instruments is alarmingly high. This multicenter study evaluated the effectiveness of instrument calibration and inter-instrument harmonization of different spectrophotometers with the same optical geometry using tooth-colored, translucent dental materials.

**Methods:**

The coordinating center (CC) spectrophotometer was calibrated using the NPL Ceram Series II set. Two sets of 10 specimens, labeled 1 to 10 and I to X (10 mm in diameter and 1 mm thick), were tested at CC and three research sites (RS1, RS2, and RS3) using the same d/8° optical geometry spectrophotometers. Calibration factors were calculated for each material and site to obtain the average calibration factors for sets 1–10, set I-X, and the combination of both. The differences among the non-corrected and corrected reflection values were calculated using CIEDE2000 (DeltaE_00_) and CIELAB (DeltaE_ab_) color difference formulas and were submitted to ANOVA and Tukey test (α = 0.05).

**Results:**

A significant decrease of color differences between non-corrected as compared to corrected measurements was recorded for all CC-RS and RS-RS comparisons. The reduction of DeltaE_00_ values between non-corrected and corrected for CC-RS1, CC-RS2, and CC-RS3 were 83.1%, 77.2%, and 73.6%, respectively. The corresponding DeltaE_00_ values for RS1-RS2, RS1-RS3, and RS2-RS3 comparisons, indirectly compared in the experiment, were 84.2%, 82.8%, and 68.5%, respectively. There was a significant reduction of DeltaE_00_ and DeltaE_ab_ color difference for all combined RS pairs and each of three RS pairs, corrected with one of two specimen sets calibration factors separately.

**Conclusions:**

Calibration and harmonization of color measurements in dentistry using tooth-colored, translucent restorative materials significantly decreased measurement discrepancies between the coordinating center and research sites and among pairs of research sites.

## Background

The interest in the optical properties of teeth and dental materials has increased over the years. A previous search in Pubmed with the keywords “color and dentistry and spectrophotometer” provided 1070 references from 1950 to May 2020 [1]. The search resulted in 485 articles published in the last three years (2021 until November 2023).

Different devices and protocols, such as visual analysis, [2] compact and hand-held spectrophotometers, [3] benchtop spectrophotometers, [4] and more recently, intra-oral scanners, [5] photocolorimetry [6] and mobile phone apps, [7] have been used for color measurements. Despite spectrophotometry being considered the gold standard for this purpose, similarly to the methods mentioned above, this instrumental analysis is susceptible to errors, which lead to measurement differences [4, 8–12]. As opposed to absolute measurements (such as height, weight, and distance), color measurements are relative. Therefore, the discrepancy among different instruments of the same optical geometry can be alarmingly high. This type of comparison would not be appropriate for instruments with different optical geometries. Calibration of the measuring instrument wavelength scale using an independent set of ceramic tiles with known reflectance or opaque white ceramic tile and black tile/trap (paired with each instrument) is recommended to reach higher reliability of measurements.

Translucent tooth-colored materials were suitable as calibration tiles [13] and for harmonizing reflection spectra measurements recorded with different spectrophotometers of the same optical geometry [14]. However, there is no information about the effect of applying calibration factors calculated from a set of realistic translucent tooth-colored specimens to harmonize the spectral wavelength of unknown specimens. This multicenter study evaluated the effectiveness of instrument calibration and inter-instrument harmonization of color measurements of spectrophotometers with the same optical geometry using tooth-colored, translucent dental materials. If validated, the proposed method of applying calibration factors calculated from a distinct set of specimens for reflection spectra measurements harmonization could be used as a reference for further modifications of ISO standards. The research hypotheses were that there would be a significant reduction of color differences (ΔE_00_, ΔE_ab_) among:

1. Coordinating Center (*CC*) and research sites (*RS*) for non-corrected (*NC*) and corrected (*CO*) measurements;
2. *RS* pairs for non-corrected (*NC*) and corrected (*CO*) measurements;
3. All *RS* pairs combined and each of three *RS* pairs, for non-corrected (*NC*) measurements, corrected (*CO*), corrected with one of two specimen sets calibration factor (*CF*) separately (*CO* 1–10 and *CO* I-X).

## Methods

Two sets of 10 tooth-colored restorative material specimens each, labeled from 1 to 10 and I to X (Table 1), were used in this study (Table 1). Specimens were prepared at the coordinating center – CC (Table 2), from commercially available pre-sintered blocks and were sintered to reach final dimensions (12.5 mm × 10 mm, 1 mm thick). After light polymerization, resin-based disks (10 mm in diameter, 1 mm thick) were finished with # 400 SiC paper under water cooling using a grinder/polisher (Ecomet 6, Buehler, Lake Bluff, IL) at 150 rpm. A 40-second polishing was performed with Enhance PoGo polisher (Dentsply Sirona, Charlotte, NC) mounted in a low-speed handpiece (maximum 15,000 rpm) with light hand pressure [4]. 

**Table 1:** Tested materials, manufacturer, and lot number [4].

Code	Material	Manufacturer	Lot
	***Set 1–10***		
1	Filtek Supreme Ultra	3 M ESPE, St. Paul, MN	N542570
2	Lava Ultimate	3 M ESPE	N502016
3	CeraSmart	GC America, Alsip, IL	140,206
4	IPS e.max CAD	Ivoclar Vivadent, Amherst, NY	R08350
5	Vita Mark II	Vita North America, Yorba Linda, CA	34,050
6	Vita Suprinity PS	Vita North America	39,311
7	Vita Enamic	Vita North America	44,670
8	DD Bio ZX2	Dental Direkt, Spenge, Germany	5,031,531,013
9	DD Cube X2	Dental Direkt	8,031,535,007
10	Origin Beyond	B&D Dental, West Valley City, UT	NA
	***Set I-X***		
I	Zenostar Translucent	Ivoclar Vivadent	NA
II	BruxZir HT	Glidewell Laboratories, Newport Beach, CA	NA
III	NexxZr T	Sagemax Bioceramics, Federal Way, WA	NA
IV	Lava Plus	3 M ESPE	NA
V	Vita YZ HT	Vita North America	NA
VI	Origin Live HT	B&D Dental	NA
VII	Nacera Pearl 1	DOCERAM Medical Ceramics, Dortmund, Germany	NA
VIII	DD Cube X2	Dental Direkt	NA
IX	Katana UT	Kuraray Noritake, Osaka, Japan	NA
X	Katana HT	Kuraray Noritake	NA

The translucency parameter (TP) was calculated as the difference in the color of the same specimen against white (L*=96.0, a*=-0.6 and b*=2.0) and black (L*=23.2, a*=0.0 and b*=-0.5) ceramic tiles – backing. The mean (SD) TP of the 1–10 set was 16.3 (4.6), ranging from 9.3 to 21.1, while the corresponding values for I-X set were 9.7 (0.7), ranging from 8.5 to 10.8.

The CC spectrophotometer Ci7600 (X-Rite, Grand Rapids, IL) was calibrated using 12 opaque ceramic tiles with known reflection and L*a*b* values provided by the National Physical Laboratory, the UK National Metrology Institute (NPL Set 1241 Ceramic Colour Standards – Series II, Gloss Set 0635, Certificate of Calibration No. 01225, CERAM Researches, Teddington, UK). Five measurements were performed for each tile, presenting an average of three consecutive readings without replacement [4]. 

The following configuration: CIE D65 standard illuminant, d/8°, 2° 1931 standard observer, specular component included (SCI), UV component included, and small area view (SAV) aperture (6 mm in diameter), was used to record reflection values from 380 to 750 nm, at 10 nm intervals. A final wavelength-dependent Calibration Factor, *CF*_(λ)_, computed as the mean value across all data sets, was calculated from the Individual Calibration Factor, *CF*_(λ)_, for each wavelength and each of the 12 calibration tiles [4]. Once the mean *CF*_(λ)_ was determined, the *CC* Reflectance Calibration values, *R*_*C*(λ)_, were computed as follows: [15]

Eq. 1 $${R_C}_{(\lambda )} = {R_{\left( \lambda \right)}} \times C{F_{(\lambda )}}$$

*R*_*C*_ corresponds to calibrated reflectance measurements, and *R*_(λ)_ corresponds to non-calibrated reflectance measurements at *CC*, respectively [15].

The *CC* spectrophotometer exhibited outstanding accuracy before and after calibration, with ΔE_00_ of 0.28 (0.10) and 0.26 (0.16), respectively, and the corresponding ΔE_ab_ values of 0.43 (0.20) and 0.40 (0.31), respectively.

Spectral reflection values of tooth-colored specimens were measured against the white calibration tile. Three measurements were obtained for each specimen, and the mean value was used for harmonization [4]. Specimens were then sent to three research sites (*RS*) in Brazil, where the reflectance spectra of each specimen were measured using contact-type d/8° spectrophotometers (Table 2), using the identical method and setup for the *CC* spectrophotometer.

**Table 2:** Research sites and spectrophotometers involved in the present study

Research site	Institution	Spectrophotometer
Coordinating Center (*CC*)	University of Texas School of Dentistry at Houston, TX	Ci7600 (X-Rite, Grand Rapids, IL)
Research site 1 (*RS1*)	School of Dentistry, Institute of Science and Technology, São José dos Campos - UNESP, SP	CM2600D (Konica Minolta, Chiyoda, Tokyo, Japan)
Research site 2 (*RS2*)	Faculty of Dentistry of The University of São Paulo, SP	CM3700D (Konica Minolta)
Research site 3 (*RS3*)	Department of Mechanical Engineering of The Federal University of Santa Catarina, SC	CM3600A (Konica Minolta)

Data on non-corrected Reflectance Spectra, *R*_(λ)_, for each material and each *RS*, were used to calculate *CF*_(λ)_ based on *R*_*C* (λ)_ values recorded at *CC* [4]. *CF*_(λ)_ values were calculated for each material and site to obtain the average *CF* for sets 1–10, set I-X, separately, and their combination. The mean values for each set, separately, and the combination for all 20 specimens (at each wavelength) were computed as *CF* for each *RS*. Data on the reflectance spectra of each *RS* were then corrected using Eq. 1.

Non-corrected (*NC*) and corrected (*CO*) reflection values from *CC* and *RS*s were converted into CIEDE2000 [16] and CIELAB [17] values, and respective color differences were calculated [4]. CIELAB color differences were used to facilitate comparisons with previous studies. The ΔE_00_ and ΔE_ab_ comparisons of *NC* and *CO* values for all *CC*-*RS* and *RS-RS* pairs, for each specimen set and their combination, were performed using Two-way ANOVA and Tukey test (JASP, 0.18.0, Department of Psychological Methods, University of Amsterdam, Amsterdam, The Netherlands), at α = 0.05. In addition, color differences were interpreted through corresponding visual thresholds: ΔE_00_ ≤ 0.8 and ≤ 1.8 (CIEDE2000 50:50% perceptibility – PT and 50:50% acceptability threshold – AT, respectively), and corresponding ΔE_ab_ ≤ 1.2 (PT) and ΔE_ab_ ≤ 2.7 (AT) [18]. Color differences above the AT were categorized as mismatch type [a] or moderately unacceptable (> AT, ≤ AT × 2), mismatch type [b] or clearly unacceptable (> AT × 2, ≤AT × 3), and mismatch type [c] or extremely unacceptable (> AT × 3) [19].

## Results

The results of the Analysis of Variance showed a significant reduction of ΔE_00_ and ΔE_ab_ values upon harmonization (*p* < 0.05). The comparisons of color differences between *CC* and non-corrected (*NC*)/corrected (*CO*) *RS*s are presented in Table 3.

**Table 3:** Mean ΔE_00_ and ΔE_ab_ values and standard deviation of paired Coordinating Center (*CC*) and each research site (*RS*) for non-corrected (*NC*) and corrected (*CO*) measurements

	ΔE_00_		ΔE_ab_	
Pair	*NC*	*CO*	*NC*	*CO*
*CC-RS1*	8.3 (1.1) A, a	1.4 (0.7) B, a	11.2 (1.2) A, a	1.8 (1.1) B, a
*CC-RS2*	6.6 (1.4) A, b	1.5 (0.7) B, a	10.0 (1.9) A, b	2.1 (1.4) B, a
*CC-RS3*	1.9 (0.4) A, c	0.5 (0.2) B, b	2.6 (0.6) A, c	0.6 (0.4) B, b

Different upper-case letters in the rows and lower-case letters in columns represent significant differences according to the Tukey test (*p* < 0.05). *Calibration Factor was calculated from the average wavelength of 20 specimens*

The mean reduction of corrected color differences compared to non-corrected ones was 77.6% (CIEDE2000) and 80% (CIELAB). The CIEDE2000 color difference reduction of 83.1; 77.2; and 73.6% was recorded for *CC-RS1*, *CC-RS2*, and *CC-RS3* comparisons, respectively. The corresponding CIELAB color difference reduction was 83.9, 79.0, and 76.9%, respectively.

The harmonization of pairs of research sites (*RS*) and corresponding comparisons among them are shown in Table 4. The ΔE_00_ and ΔE_ab_ values were calculated from non-corrected (*NC*) and corrected (*CO*) wavelengths.

**Table 4:** Mean values and standard deviation of ΔE_00_ and ΔE_ab_ of paired research site (*RS*) for non-corrected (*NC*) and corrected (*CO*) measurements

	ΔE_00_		ΔE_ab_	
Pair	*NC*	*C* *O*	*NC*	*CO*
*RS1-RS2*	16.5 (1.4) A, a	2.6 (1.4) B, a	21.1 (2.9) A, a	3.6 (2.2) B, a
*RS1-RS3*	11.4 (1.2) A, b	1.9 (1.0) B, ab	13.8 (1.6) A, b	2.3 (1.4) B, a
*RS2-RS3*	5.2 (0.7) A, c	1.6 (0.9) B, b	7.4 (1.3) A, c	1.5 (0.7) B, b

Different upper-case letters in the row for each color difference and small letters in columns represent significant differences, according to the Tukey test (*p* < 0.05). *The Calibration Factor was calculated from the average wavelength of 20 specimens*

The mean reduction of corrected color differences compared to non-corrected ones was 82.1% (CIEDE2000) and 78.5% (CIELAB). The CIEDE2000 color difference reduction of 84.2; 82.8; and 68.5%, was recorded for *RS1-RS2*, *RS1-RS3* and *RS2-RS3* comparisons, respectively. The corresponding CIELAB color difference reduction was 82.9, 83.4, and 80.0%, respectively.

The result of the Analysis of Variance for ΔE_00_ and ΔE_ab_ of each specimen set (1–10 and I-X) for paired *RS* and *CO* wavelengths, calculated separately from the two sets, showed the significance of paired *RS* and for the interaction of specimen set *vs. CO* (*p* < 0.001, for both). Comparisons of ΔE_00_ and ΔE_ab_ for each specimen set, for all combined *RS* pairs, calculated from the *CO* 1–10 and *CO* I-X separately, are presented in Table 5.

**Table 5:** The mean values and standard deviation of ΔE_00_ and ΔE_ab_ for each specimen set (1–10 and I-X) and all RS pairs, calculated separately from *CO* 1–10 and *CO* I-X

	ΔE_00_		ΔE_ab_	
Set	*CO* 1–10	*CO* I-X	*CO* 1–10	*CO* I-X
1–10	1.5 (1.2) a	2.1 (1.1) a	2.0 (1.7) a	2.9 (1.7) a
I-X	2.6 (1.2) a	1.4 (1.4) b	3.3 (1.9) a	2.1 (2.0) b

Different lower-case letters in the rows represent significant differences according to the Tukey test (*p* < 0.05). *Calibration Factor has been calculated from the average wavelength of 10 specimens, separately, CO* 1–1 *and CO* I-X

The comparisons of ΔE_00_ and ΔE_ab_ values of the same specimens set, calculated from the average wavelength of 10 specimens, separately, *CO* 1–10 and *CO* I-X, showed similar and comparable values for set 1–10. For the set I-X, comparable but significantly different values were obtained with *CO* 1–10 and *CO* I-X for ΔE_ab_ and ΔE_00_ values.

Comparisons of ΔE_00_ and ΔE_ab_ of paired research site (*RS*), for each specimen set (1–10 and I-X), calculated from non-corrected (*NC*) wavelengths and calibration factor calculated from the average wavelength of 10 specimens, separately, *CO* 1–10 and *CO* I-X, are presented in Table 6.

**Table 6:** Mean values and standard deviation of ΔE_00_ and ΔE_ab_ of paired research sites (*RS*) for each specimen set (1–10 and I-X), calculated from non-corrected (*NC*) wavelengths and calibration factor calculated from the average wavelength of 10 specimens, separately, *CO* 1–10 and *CO* I-X

Pair	Set	ΔE_00_			ΔE_ab_		
		*NC*	*CO* 1–10	*CO* I-X	*NC*	*CO* 1–10	*CO* I-X
*RS1-RS2*	1–10 I-X	15.8 (1.6) b 13.8 (2.7) b	2.2 (1.7) a 3.7 (1.2) a	3.0 (1.3) a 2.1 (1.8) a	22.2 (2.0) b 20.0 (3.3) b	3.0 (2.3) a 4.9 (2.0) a	4.3 (2.0) a 3.1 (2.0) a
*RS1-RS3*	1–10 I-X	10.6 (0.9) b 9.6 (1.8) b	1.4 (0.9) a 2.3 (0.9) a	1.9 (0.7) a 1.6 (1.1) a	14.2 (1.0) b 13.3 (2.1) b	1.8 (1.3) a 3.0 (1.6) a	2.4 (1.1) a 2.2 (1.7) a
*RS2-RS3*	1–10 I-X	5.4 (0.8) b 4.4 (0.9) b	0.9 (0.6) a 1.6 (0.3) a	1.3 (0.6) a 0.6 (0.6) a	8.1 (1.2) b 6.9 (1.3) b	1.3 (0.9) a 2.0 (0.5) a	2.0 (0.9) a 0.9 (0.9) a

Different lower-case letters in the row for each color difference represent no significant difference, according to the Tukey test (*p* < 0.05). *NC – non-corrected values; calibration factor calculated from the average wavelength of 10 specimens, separately, CO* 1–10 *and CO* I-X

ΔE_00_ and ΔE_ab_ values of each set of specimens showed no differences between ΔE_00_ calculated from the average wavelength of specimens 1–10 and I-X. The same results were observed for ΔE_ab_ values.

## Discussion

Industry/profession-specific harmonization using translucent tooth-colored dental materials of different spectrophotometers with the same optical geometry has been proven effective [1, 4]. Hence, the first research hypothesis has been accepted. Two of three *CC-RS* pairs exhibited high non-corrected ΔE_00_ and ΔE_ab_, and the remainder *CC-RS3* pair presented comparable values (Table 3). Higher non-corrected ΔE_00_ and ΔE_ab_ total color differences are possibly due to the lack of a more frequent calibration of the instruments of research sites tested in the study. Comparable ΔE_00_ and ΔE_ab_ values were obtained for *CC-RS* pairs upon harmonization.

In the present study, a significant decrease of ΔE_00_ and ΔE_ab_ color differences among different sites was achieved upon correcting reflection values, using each set of tooth-colored dental materials as calibration tiles or the combination of them. Despite the TP difference between specimens set, similar reduction of ΔE_00_ and ΔE_ab_ color differences were observed (Table 4), which confirmed previous results using the same protocol and specimens [4]. Both set of specimens had previously been tested in other studies and had proved to be effective as calibration targets for harmonization of color measurements [1, 4]. This result could be achieved because plastics and ceramics fulfill some of the required properties of a calibration target, such as high and constant reflectance over small variations of angles of incidence, durability, [13] stability, and easy handling [20]. 

Previous studies had harmonized the spectral wavelength of one set of specimens itself [1, 4]. As far as it is known, this study is the first to use translucent tooth-colored materials to calculate calibration factors and harmonize reflection spectra measurements of another set of specimens. For set 1–10, similar ΔE_00_ and ΔE_ab_ color differences were obtained for combined *RS* pairs, with the calibration factor calculated from reflection spectra of set 1–10 compared to set I-X (Table 5). The same result was observed for each *RS* pair. For set I-X, comparable but significantly different ΔE_00_ and ΔE_ab_ color differences were observed for combined *RS* pairs for the comparison between calibration factor calculated from reflection spectra of set 1–10 compared to set I-X. Conversely, similar ΔE_00_ and ΔE_ab_ color differences were obtained for each *RS* pair (Table 6).

When interpreting non-corrected ΔE_00_ total color differences, 100% of *CC-RS* pairs and 100% of *RS* pairs presented values above AT. Two of three *CC-RS* pairs and two of three *RS* pairs followed into an extremely unacceptable mismatch, while the pair *RS2-RS3* corresponded to an unacceptable mismatch. Upon harmonization, 100% of ΔE_00_ and ΔE_ab_ total color differences for *CC-RS* pairs were below AT. An excellent match was noted for the *CC-RS3* pair, while an acceptable match was registered for the *CC-RS1* and *CC-RS2* pairs. For *RS* pairs, ΔE_00_ and ΔE_ab_ total color differences for *RS1-RS2* and *RS1-RS3* pairs corresponded to a moderately unacceptable mismatch, while *RS2-RS3* corresponded to an acceptable match.

For combined *RS* pairs, ΔE_00_ and ΔE_ab_ total color differences for *CO* 1–10 and *CO* I-X corresponded to acceptable match and moderately unacceptable mismatch for specimens set 1–10. For the set I-X, acceptable match and moderately unacceptable mismatch were observed with *CO* I-X and *CO* 1–10, respectively.

Non-corrected ΔE_00_ and ΔE_ab_ values were calculated for each *RS* pair and each set of specimens, 100% of values were above AT. For *RS1-RS2* and *RS1-RS3* pairs, color differences corresponded to an extremely unacceptable mismatch, while for the *RS2-RS3* pair, it corresponded to a clearly unacceptable mismatch. After harmonization, an excellent match of ΔE_00_ and ΔE_ab_ total color differences was observed for *RS2-RS3* set I-X calculated with *CO* I-X. Five of 11 ΔE_00_ total color differences were categorized as acceptable match, five as moderately unacceptable mismatch, and one as clearly unacceptable mismatch. Six of eleven ΔE_ab_ total color differences corresponded to an acceptable match, while five corresponded to a moderately unacceptable mismatch.

The indirect harmonization among research sites by comparisons with a master laboratory, using calibration factors for an unknown set of specimens, proved effective as the grades of ΔE_00_ and ΔE_ab_ total color differences shifted from extremely unacceptable mismatch (non-corrected wavelengths) to acceptable match. It also highlighted the importance of harmonizing the wavelength spectra to provide consistent and comparable color measurements. This could be obtained with a small number of specimens, which is important to keep this protocol as easy and practical as possible.

Using industry/profession-specific clinically relevant translucent tooth-colored materials as calibration tiles in a standardized protocol, in addition to the methodologies of previous studies [1, 4], is a valuable tool for significantly reducing color differences among different sites, thus facilitating multicenter studies and communication. However, it should be used with care because spectrophotometry is designed for analysis of flat surfaces, which is not suitable for color measurement of curved surfaces of natural tooth and restorations. Furthermore, special attention should be given to the preparation of specimens used as calibration tiles to achieve and maintain materials color stability.

## Conclusions

Within the limitations of this study, it was concluded that:

1. Harmonization of reflection spectra measurements using tooth-colored, translucent restorative materials resulted in a significant reduction of color differences (ΔE_00_, ΔE_ab_) among all *Coordinating center – research site* pairs;
2. In addition, the method resulted in a significant decrease in color differences (ΔE_00_, ΔE_ab_) among *research site* pairs;
3. Using the Calibration Factors of each of the two specimen sets separately (1–10 and I-X) enabled a significant reduction of color differences (ΔE_00_, ΔE_ab_) among all *research site* pairs.

## Data Availability

No datasets were generated or analysed during the current study.

## Abbreviations

*CC*: Coordinating center
*RS*: Research sites
*CF*: Calibration factor
*NC*: Non-corrected
*CO*: Corrected
SCI: Specular component included
SAV: Small area view
*CF*_(λ)_: Individual Calibration Factor
*R*_*C*(λ)_: Reflectance Calibration value
*R*_*C*_: Calibrated reflectance measurement
*R*_(λ)_: Non-calibrated reflectance measurement
PT: CIEDE2000 50:50% Perceptibility threshold
AT: CIEDE2000 50:50% Acceptability threshold
